# Down-Regulation of the RNA Editing Enzyme ADAR2 Contributes to RGC Death in a Mouse Model of Glaucoma

**DOI:** 10.1371/journal.pone.0091288

**Published:** 2014-03-07

**Authors:** Ai Ling Wang, Reed C. Carroll, Scott Nawy

**Affiliations:** Department of Ophthalmology and Visual Sciences, Albert Einstein College of Medicine, Bronx, New York, United States of America; Dalhousie University, Canada

## Abstract

Glaucoma is a progressive neurodegenerative disease of retinal ganglion cells (RGCs) associated with characteristic axon degeneration in the optic nerve. Excitotoxic damage due to increased Ca^2+^ influx, possibly through NMDA-type glutamate receptors, has been proposed to be a cause of RGC dysfunction and death in glaucoma. Recent work has found that expression of another potentially critical receptor, the Ca^2+^-permeable AMPA receptor (CP-AMPAR), is elevated during various pathological conditions (including ALS and ischemia), resulting in increased neuronal death. Here we test the hypothesis that CP-AMPARs contribute to RGC death due to elevated Ca^2+^ influx in glaucoma. AMPA receptors are impermeable to Ca^2+^ if the tetrameric receptor contains a GluA2 subunit that has undergone Q/R RNA editing at a site in the pore region. The activity of ADAR2, the enzyme responsible for this RNA editing, generally ensures that the vast majority of GluA2 proteins are edited. Here, we demonstrate that ADAR2 levels decrease in a mouse model of glaucoma in which IOP is chronically elevated. Furthermore, using an *in vitro* model of RGCs, we find that knockdown of ADAR2 using siRNA increased the accumulation of Co^2+^ in response to glutamate, and decreased the rectification index of AMPA currents detected electrophysiologically, indicating an increased Ca^2+^ permeability through AMPARs. The RGCs in primary culture also exhibited increased excitotoxic cell death following knock down of ADAR2. Furthermore, cell death was reversed by NASPM, a specific blocker for CP-AMPARs. Together, our data suggest that chronically elevated IOP in adult mice reduces expression of the ADAR2 enzyme, and the loss of ADAR2 editing and subsequent disruption of GluA2 RNA editing might potentially play a role in promoting RGC neuronal death as observed in glaucoma.

## Introduction

Glaucoma is the second leading cause of blindness overall, and the leading cause of blindness in the African American community [1]. There is no single cause of glaucoma, as glaucoma itself is not a single disease, but rather a group of diseases characterized by optic nerve degeneration. The optic nerve is formed by the axons of retinal ganglion cells (RGCs), and it is this type of cell that is primarily targeted in glaucoma [2]. Preventing RGC death is therefore a clear goal for treating patients with glaucoma [3]. Years of intensive research on the part of both clinical and basic research scientists have led to the understanding that a number of different underlying mechanisms contribute to this disease. These include: neurotropic factor deprivation [4], [5], axonal transport failure [6], [7], activation of intrinsic and extrinsic apoptotic signals [8], [9], oxidative stress [10], hypoperfusion/ischemia of the anterior optic nerve [11], glial cell activation [12], [13] and glutamate excitotoxicity [14], [15]. While it is understood that all of these factors contribute to the loss of RGCs in glaucoma, this study focused on glutamate excitotoxicity as an underlying cause of RGC death.

Glutamate is the predominant excitatory neurotransmitter in the retina. It is released by all of the cell types that comprise the primary flow of visual information, including photoreceptors, bipolar cells, and RGCs. Elevation of endogenous glutamate and activation of glutamate receptors have been shown to contribute to a variety of acute and chronic neurological disorders, including stroke, Alzheimer’s disease and amyotrophic lateral sclerosis (ALS) [16]. In the retina, excess glutamate has been proposed as well to underline several diseases such as retinal artery occlusion and glaucoma [17]. It was widely believed that these effects are mediated by excess glutamate and elevated activation of NMDA receptors. Excess glutamate binds to NMDA receptors in neurons, and because NMDA receptors are highly permeable to Ca^2+^, influx of this cation leads to activation of apoptotic signaling pathways [14]. However, the elevations in glutamate release remain controversial and clinical trials of NMDA antagonists in glaucoma have so far been disappointing [18]. Recent evidence suggests that increased Ca^2+^ influx through AMPARs, independent of increased glutamate release or NMDAR activation, can also contribute to excitotoxic cell death [19].

AMPA receptors, which are tetramers of glutamate receptor 1–4(GluA1–4) subunits, mediate rapid excitatory synaptic transmission [20]. The presence of the edited GluA2 subunit critically determines AMPA properties including Ca^2+^ permeability, as AMPA receptors lacking edited GluA2 subunits are permeable to Ca^2+^ [21]. Ca^2+^ influx through CP-AMPARs is crucial in several forms of synaptic plasticity and has also been linked to cell death associated with neurological diseases. AMPARs that contain the GluA2 subunit are impermeable to Ca^2+^. However, this requires that the GluA2 transcript is edited at the Q/R site, resulting in substitution of an arginine residue for glutamine. The enzymes responsible for RNA editing are termed “adenosine deaminases acting on RNA” (ADARs), and three structurally related ADARs (ADAR1 to ADAR3) have been identified in mammals. Thus, injury or diseases that result in decreased expression of the ADAR family could be expected to result in increased Ca^2+^ influx into neurons through AMPARs that are normally impermeable to Ca^2+^. For example, in motor neurons of ALS patients and cortical neurons following forebrain ischemia, expression levels of ADAR2 is reduced and neuronal death has been attributed to increased Ca^2+^ permeability through the unedited GluA2-containing receptors.

The purpose of the work is to understand the pathophysiological role of ADAR2 and CP-AMPA receptors in RGCs using an experimentally induced glaucoma model. Our data suggest that in an adult mouse model in which IOP has been chronically elevated, there is a selective reduction in the expression of the ADAR2 enzyme, and disruption of the RNA editing of the GluA2 subunit, a molecular change that increases Ca^2+^ influx through AMPA receptors, leading to selective neuronal death.

## Materials and Methods

### Animals

A total of 68 female 10-week old C57BL/6mice were utilized in this study. All animals were treated in accordance with the ARVO Statement for the Use of Animals in Ophthalmic and Vision Research, using protocols approved and monitored by the Albert Einstein College of Medicine of Medicine Animal Care and Use Committee.

### Microbead Surgeries and IOP Measurements

For microbead surgeries, one eye was injected with sterile 6 µm polystyrene microbeads in a 3×10^6^ microbeads/mL solution (Polysciences, Inc. Warrington PA), and the other eye was injected with an equivalent volume of sterile physiologic saline (Fisher Scientific, Fair Lawn, NJ). As such, each animal serves as its own control. Before injections, animals were anesthetized with isoflurane (Minrad Inc., Bethlehem, PA), which was controlled by a pump that allows continuous administration for up to 12 hours. Pupils were dilated with 1% tropicamide ophthalmic solution (Bausch & Lomb, Tampa, FL), and anesthetic drops (0.5% proparacaine hydrochloride; Bausch & Lomb) were applied to each eye. Injections were made using a 33-gauge needle (Hamilton Corporation) inserted parallel to the iris. The 33-gauge needles were beveled on three sides to facilitate injection through the cornea and provided the easiest transit through the corneal stroma. A tunneled injection was made with the needle running parallel to the anterior surface of the iris to minimize the risk of iris trauma. The fluid was slowly injected over 45 second, and the empty cannula was kept in place for 2 min to minimize eflux of injected material.

Intraocular pressure (IOP) measurements were made in both eyes with the TonoLab tonometer (TioLat, Helsinki, Finland) under combined topical anesthesia with 0.5% proparacaine hydrochloride eyedrops. IOP was measured in an awake state. IOP was measured prior to bead injection (baseline), 2 days after injection, and in all animals 3–7 days after injection thereafter until 6 weeks after injection.

### Real Time RT-PCR

Total cellular RNA from mouse retinas and cultured retinal cells was isolated and purified (Qiagen, Germantown, MD). Samples of the total starting RNA were analyzed by capillary electrophoresis (Agilent Technologies, Palo Alto, CA) to assess the degree of purification. Real time quantitative RT-PCR (qRT-PCR) was done using the SYBR-Green dye binding method implemented on an Applied Biosystems 7900 genetic analyzer at the Genomics Facility at the Albert Einstein College of Medicine. Validated primers for each gene of interest were designed for each target mRNA (Table 1). Optimization of primers and determination of the input cDNA levels were done to ensure appropriate cycle time response. Relative expression was calculated from the differences in cycle time of an internal standard (GAPDH) compared to the target mRNA. All qRT-PCR reactions used 40 ng of cDNA produced as described above and were run with two sets of duplicates from three to five different samples per group.

**Table 1:** Primers for real-time RT-PCR.

**Adar2**
5′- CCA GTG GAG ATG GGA AGA TAG A -3′
Sense
5′- ACA TGG AAA GCC AGA TGT AGT G -3′
Anti-sense
**GluA2**
5′- TAG ACT CTG GCT CCA CTA AAG A -3′
Sense
5′- GTA GTC CTC ACA AAC ACA GAG G -3′
Anti-sense
**Rest**
5′- AGA GAG GCT GAC CTG CTT AAT A-3′
Sense
5′- CGT TCT TCT TCC CAG ACA CAT C-3′
Anti-sense
**GAPDH**
5′- TCA ACA GCA ACT CCC ACT CTT CCA-3′
Sense
*5* ′*- ACC CTG TTG CTG TAG CCG TAT TCA-3*′
*Anti-sense*

### Western Blot

Mouse retinal tissues were dissected and pooled from six eyes, lysed in buffer (20 mM HEPES, pH 7.0, 10 mM KCl, 2 mM MgCl_2_, 0.5% Nonidet P-40, 1 mM Na_3_VO_4_, 1 mM PMSF, and 0.15U ml^–1^ aprotinin) and homogenized. Protein concentrations were determined using the Bradford colorimetric assay. Thirty micrograms of each protein lysate were loaded in each lane in sample buffer (2% SDS, 10% glycerol, 0.001% bromophenol blue, 1% DTT, and 0.05 M Tris-HCl, pH 6.8), separated on 10% SDS-PAGE (Invitrogen), and transferred to a PVDF membrane (Millipore, Temecula, CA). The blots were blocked with 5% nonfat milk in PBS and incubated with specific rabbit polyclonal antibody against ADAR2 and β-actin, followed by peroxidase-conjugated goat anti-rabbit IgG_2a_ (Millipore) and the enhanced chemoluminescence detection system (Amersham Biosciences, Arlington Heights, IL).

### Immunohistochemistry

Enucleated eyes were fixed in 2% wt/vol paraformaldehyde in 0.01 M phosphate buffered saline (PBS; pH 7.4) at 4°C overnight. Six animals were used for each group. Immunohistochemistry was performed on paraffin sagittal sections of retina for ADAR2 (1∶200, Abcam ab64830), following by secondary antibody. As a negative control, sections were treated in the same manner, except that incubation with primary antibody was omitted.

### TUNEL Labeling

Apoptosis was detected using the terminal deoxytransferase-mediated dUTP nick end-labeling (TUNEL) in situ apoptosis detection kit (Roche Diagnostics, Mannheim, Germany), according to the manufacturer’s instructions. Briefly, paraffin cross-sections were deparaffinized and rehydrated. The tissue samples were incubated for 6 min at room temperature with proteinase K working solution. After PBS washing, tissues were treated with TUNEL reaction mixture and incubated for 60 min at 37°C in a humidified atmosphere. Slides were then rinsed three times in PBS at room temperature for 5 min and counterstained with DAPI. Only TUNEL-positive cells in retinal ganglion cell layer were counted. For each time point, eight eyes were used for statistical analysis. The number of TUNEL positive cells per eye was calculated from the average number of four central retinal sections.

### Preparation of Cell Cultures

Cells were prepared as described previously [22]. Briefly, retinas were isolated from newborn (postnatal day 0) rats after cryoanesthesia and were incubated for 45 min at 37°C in DMEM with HEPES (Mediatech, Manassas, VA), supplemented with 6 U/ml papain (Worthington, Freehold, NJ) and 0.2 mg/ml cysteine. Papain was then inactivated by replacing the enzyme solution with complete medium composed of DMEM, 5 mm HEPES, 0.1% Mito^+^ serum extender (Collaborative Research, Bedford, MA), 5% heat-inactivated fetal calf serum, 0.75% penicillin–streptomycin–glutamine mix (Invitrogen, Carlsbad, CA). Osmolarity was adjusted to 300 mOsm by addition of distilled water. Retinas were triturated through a fire-polished Pasteur pipette, plated onto glass coverslips pretreated with poly-d-lysine (0.1 mg/ml), and maintained in complete medium supplemented with 15 mm KCl. At 72 h after plating, cells were treated with the antimitotics 5-fluoro-2-deoxyuridine (0.01 mg/ml) and uridine (0.026 mg/ml) for 24 h. Subsequently, every second day, 50% of the culture medium was exchanged for fresh medium. Cells were used for siRNA experiments within 14 days.

### Patch Clamp Recording

Recordings from cultured RGCs were obtained with an Axopatch 200B using Axograph acquisition software and digitized with an ITC-18 interface (HEKA). Recording pipettes were pulled from borosilicate glass (WPI) by a two-stage vertical puller (Narishige) and filled with a K^+^ gluconate-based solution (125 mM) that also contained 10 mM KCl, 10 mM EGTA, 10 mM HEPES, 4 mM ATP and 1 mM GTP (pH 7.4 by KOH) and 0.3–0.5 mM spermine, which was diluted into the internal solution immediately before experiments. Pipette resistances were typically 3.5–5 MΩ. Holding potentials were corrected for a 10 mV junction potential, but series resistance, typically measuring 15–20 MΩ, was not compensated for. The composition of the external solution was 147 mM NaCl, 2 mM KCl, 2 mM CaCl_2_, 1.5 mM MgCl_2,_ 10 mM HEPES (pH 7.4). To block inhibitory input, 100 µM picrotoxin was added to the external solution on the day of the experiment. Analysis was performed off line using Axograph X and Kaleidagraph (Synergy Software) software. Rectification ratios were measured in the following way: I–V relations of AMPA (200 µM) responses were measured for each cell at −60, −10 and 40 mV. Responses were first normalized to responses obtained at −60 mV (A line was then fit to the region of the I–V between −60 mV and −10 mV. The rectification ration was calculated as the ratio of the measured and extrapolated currents at +40 mV. This method does not rely on any assumption about reversal potentials, as do 2-point measurements of rectification ratio, since this method essentially normalizes the size of the measured current to the predicted linear current, regardless of the specific reversal potential for that cell.

### siRNA Transfections

The siRNA oligonucleotides for ADAR2 were predesigned and synthesized by Invitrogen. The following SiRNA for ADAR2 (Cat. # AM16708) was designed to target rat ADAR2 transcripts (NM_001111055.1, NM_001111056.1, NM_001111057.1 and NM_012894.2). RNA duplexes (20 nM) were transfected into cells using Lipofectamine 2000 according to the manufacturer’s protocol. Briefly, cultured retinal cells were plated in 24-well plates for 5–8 days. The cell media was changed to 400 µl of DMEM without serum and antibody per well before transfection. RNAi duplex was diluted in DMEM and mixed gently with Lipofectamine 2000 mixture (Invitrogen) with a ratio 1∶1, followed by incubation for 20 min at room temperature and then was added to cell culture wells. The cells were incubated in a standard cell culture incubator for 5 h. Then the medium was changed back to normal medium for further experiments. In parallel to each experiment, knockdown of ADAR2 mRNA was confirmed by quantitative RT-PCR, western blot and immunocytochemistry. Transfections were performed in triplicate, and all experiments were repeated three times.

### Immunocytochemistry and Image Analysis

To examine the changes of ADAR2 expression in cultured retinal cells with siRNA, fixed retinal cells were incubated with an anti-ADAR2 Ab (1∶200) overnight, following by secondary antibody conjugated with Cy3. ADAR2 positive cells were imaged using a Hamamatsu Orca ER camera mounted on an inverted Nikon fluorescent microscope with a 60 Å∼ Plan Apo lens. Exposures were adjusted to ensure signals throughout the neuron fell within the linear range of the camera. Images were analyzed using Metamorph software (Molecular Devices, Sunnyvale, CA). Images were background-subtracted and thresholded to include signals more than 2-fold greater than the diffuse labeling in nuclei to highlight ADAR2 labeling. Integrated signal intensity values of fluorescence were determined for dendrites, normalized to area, and were graphed as a percent change from labeling in untreated controls. To measure the intensity of ADAR2, regions of interest were created around ADAR2 labeling and transferred to background-subtracted, thresholded ADAR2 images.

### Cobalt Permeability

The detection of Co^2+^ uptake into the cultured retinal neurons was performed with minor modifications as described by Pruss et al. (1991). Cells were rinsed twice in HEPES buffer (146 mM NaCl, 4.2 mM KCl, 0.5 mM MgCl2, 0.8 mM CaCl2, 20 mM HEPES, 55.6 mM glucose, pH 7.4) and were stimulated subsequently with agonist (in HEPES buffer containing 5 mM CoCl2) for 20 min at room temperature. The agonists used were 200 µM kainite, 500 µM APV, which blocks the desensitization at AMPA receptors (Yamada and Tang, 1993). To block the cobalt uptake pharmacologically, the selective Ca^2+^permeable AMPA receptor antagonist (25 µM NASPM) was used. Following the agonist-stimulated cobalt uptake, the cells were washed twice for 5 min and 10 min, respectively, with HEPES buffer containing 2 mM EDTA. The cobalt was precipitated by incubating with 0.12% (NH4)2S (in HEPES buffer) for 5 min. The cells were washed in HEPES buffer and then fixed in 4% paraformaldehyde containing 4% sucrose in phosphate buffered saline (PBS; 140 mM NaCl, 15 mM sodium phosphate, pH 7.4 for 20–30 min at room temperature. Enhancement of the Co^2+^ precipitate was performed by GE Healthcare’s IntensEM kit. Usually, the cells were developed sufficiently during a 50 min incubation time. Counting cells in an arbitrary field on photomicrographs was performed to determine the percentage of cobalt-stained cells.

### Statistical Analyses

Data are presented as mean± SEM with statistical differences between groups analyzed by standard Student two-tailed t-test and one way ANOVA using GraphPad Prism 5 and Kaleidagraph software. A *p* value of less than 0.05 was considered statistically significant.

## Results

### 1. The Glaucoma Model with Chronic Elevated IOP

**Figure 1:**
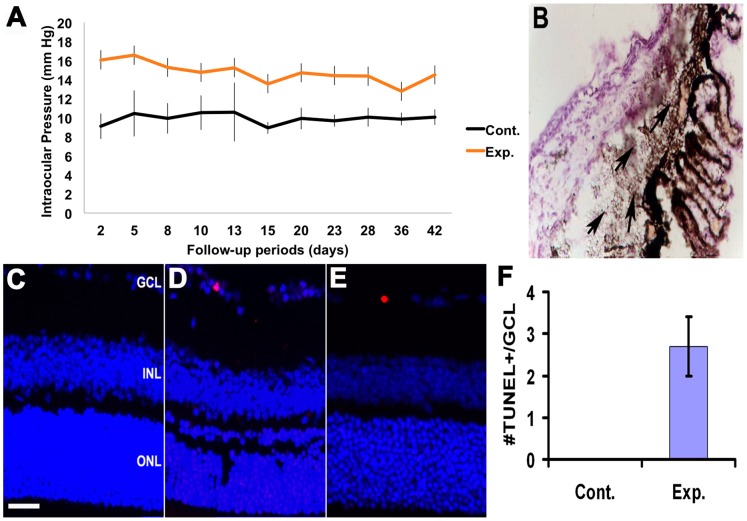
The glaucoma model with chronic elevated IOP. (A): Throughout the 6-week follow-up, IOP was elevated in the experimental eye compared with the contralateral eye (*p*<0.01, n = 14). All values are mean ± STDEV (B): Micrograph of anterior chamber from an eye injected with 2 µl microbeads. Microbeads (clear spheres) were apparent in the iridocorneal angle and clustered near the point of aqueous outflow (arrow). (C) and (D): TUNEL staining. TUNEL positive cells were shown in the RGC layer in the glaucoma model (D), but not in control group (C).

We employed a mouse model for glaucoma in which IOP is chronically elevated in adult C57bl/6 mice by intracameral injection of latex microbeads. The beads elevate IOP by blocking drainage through the trabecular meshwork. Microbead injections were made into one eye of 68 mice with sham injections in the other eye serving as controls. Microbead injections were well tolerated and did not incite a uveitic response. IOP measurements were made in both eyes in awake mice. Figure 1A demonstrates IOP profiles after injection. Throughout the 6-week follow-up, IOP was elevated in the experimental eye compared with the contralateral eye (*p*<0.01, n = 14). Histologic examination of the anterior segment of the eye after a single intraocular bead injection confirmed sequestration of the beads within the trabecular meshwork (Fig. 1B). To visualize the induction of apoptosis in the retinal ganglion cell at 6 weeks after the microbead injection, TUNEL-positive cells in the ganglion cell layer were measured (Fig. 1C and D). TUNEL-positive cells appeared in the ganglion cell layer of microbeads-injected eyes (Fig. 1D), but not in the control (Fig. 1C). The scarcity of TUNEL positive cells in GCL layer of model mice at 6 weeks suggested that RGC death was chronically induced in the microbeads-injected glaucoma model, which is consistent with the natural history of glaucoma.

### 2. ADAR2 was Decreased in Retina in a Glaucoma Model with Chronic Elevated IOP

**Figure 2:**
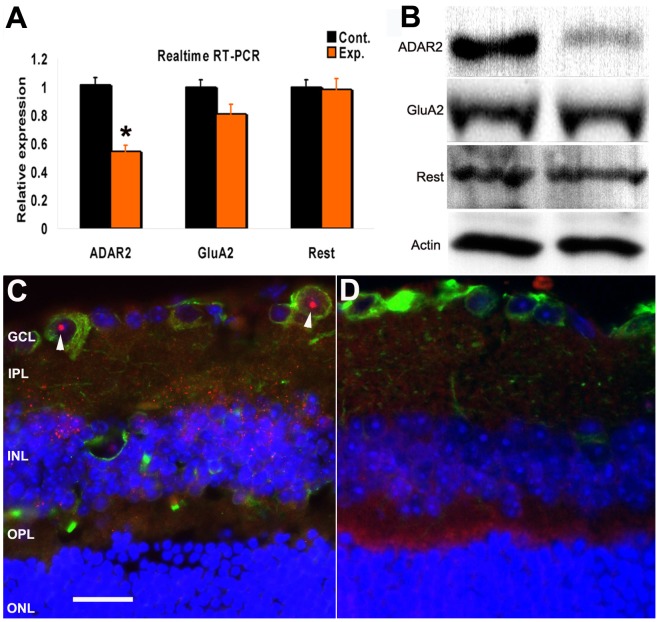
ADAR2 was decreased in retina in a glaucoma model with chronic elevated IOP. (A) In the control mouse retina, nuclear localization of ADAR2 was observed in Tuj1 positive cells. (B) In the retina of the glaucoma model, ADAR2 expression was decreased. Red: ADAR2; Tuj1: green; DAPI: blue. (C) RT-PCR data showed the expression level of ADAR2 was decreased by approximately 55% in the retina of the glaucoma model, compared with the control group (p = 0.019, n = 5). The mRNA levels of GluA2 and Rest were not changed (p>0.05, n = 5). (D). Using Western blot, the protein level of ADAR2 was decreased in the retina from the eye with elevated IOP, compared with the retina in the control eye. (Left panel, control; right panel: glaucoma group). The protein levels of GluA2 and Rest were not changed.

We sought to examine whether the elevation of IOP in glaucoma elicits any changes in glutamate receptors or glutamate receptor associated proteins, which might contribute to elevations in glutamate induced Ca^2+^ influx. Using real-time RT-PCR, we measured in control and bead injected eyes all of the subunits of AMPA, NMDA and kainate receptors, as well as additional genes that regulate GluA2 gene expression and function. These included REST, which represses GluA2 transcription and ADAR2, which mediates GluA2 RNA editing. We found a 55% reduction of ADAR2 in the bead-injected eyes (Fig. 2C). The change of ADAR2 expression was confirmed at the protein level using Western blot (Fig. 2D). Immunohistochemical labeling of ADAR2 was next examined in mouse retina. Tuj1 was used to label RGCs (Fig. 2A and B). ADAR2 was found to localize in Tuj1 positive cells in control mice (Fig. 2A) but the expression of ADAR2 was dramatically decreased in the glaucoma model.

### 3. ADAR2 siRNA in Primary Retinal Cell Culture

**Figure 3:**
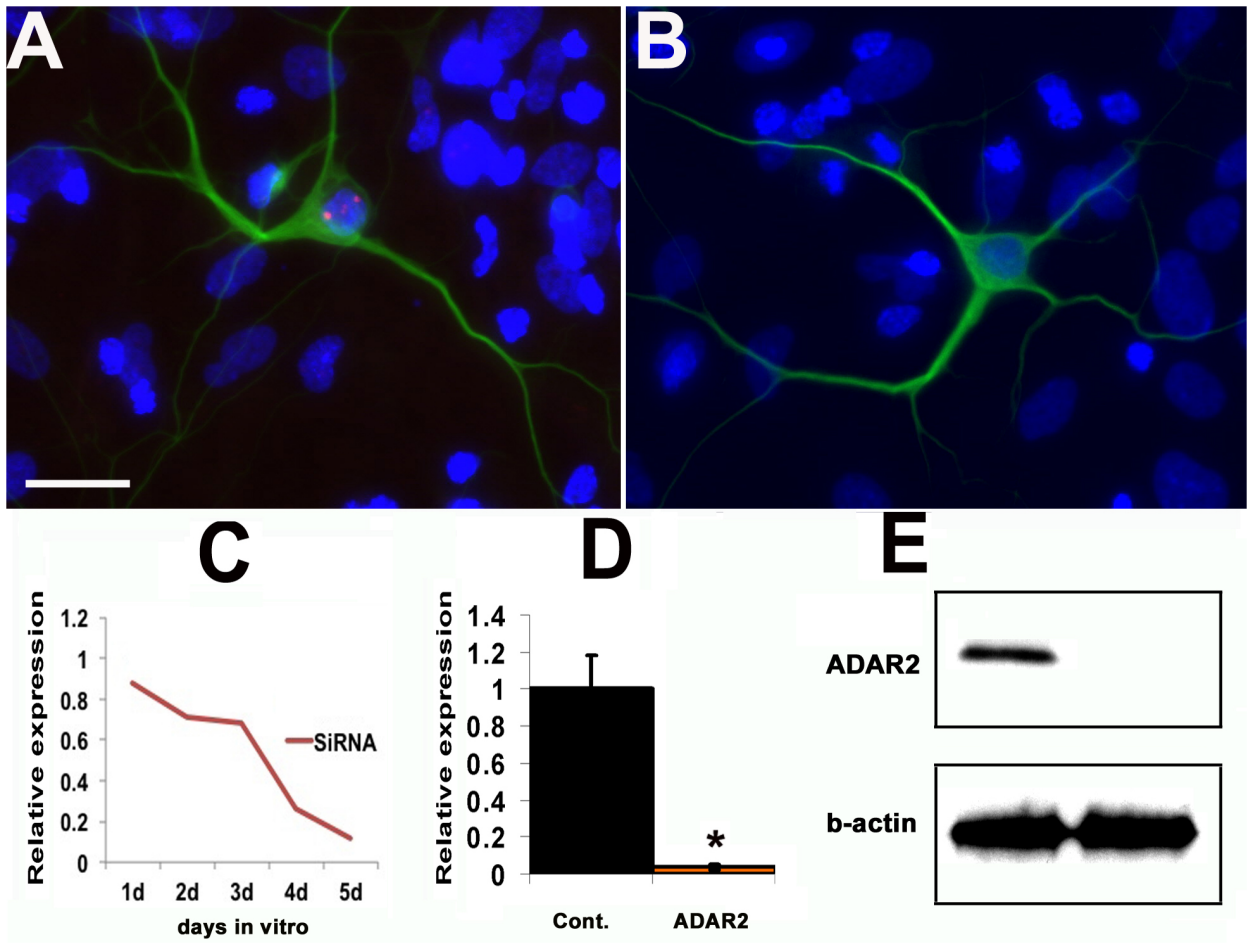
ADAR2 siRNA in primary retinal cell culture. (A) ADAR2 was consistently observed in Tuj1 positive RGCs in primary cell culture. (B) After 5 days of incubation with ADAR2 siRNA, ADAR2 expression was absent in RGCs. Red: ADAR2; Green: Tuj1; Blue: DAPI. (C) ADAR2 siRNA transfection significantly decreased ADAR2 expression in primary cell culture detected by quantitative RT-PCR, in a time dependent manner (p<0.05, n = 3). (D) ADAR2 siRNA at 5 days significantly decreased ADAR2 expression in primary cultured retina neurons detected by semi-quantitative immunocytochemistry, compared with scrambled siRNA (p = 0.036, n = 11). (E) The knockdown of ADAR2 using siRNA at 5 days was confirmed using Western blot.

To investigate ADAR2 function in retina, ADAR2 expression was knocked down using siRNA in primary cultured retinal cells. The Silencer select siRNA for ADAR2 were predesigned and synthesized by Invitrogen. RNA duplexes (20 nM) were transfected into cells using Lipofectamine 2000 according to the manufacturer’s protocol. While ADAR2 was detected immunocytochemically in Tuj1 positive retinal ganglion cells (Fig. 3A), ADAR2 labeling was largely lost with knockdown ADAR2 using siRNA (Fig. 3B, D). ADAR2 expression decreased in primary cell cultures in a time dependent manner as detected by real-time RT-PCR (Fig. 3C). Western blot analysis showed decreased ADAR2 protein in the ARAR2 siRNA-treated neurons, compared with those treated with control siRNA (Fig. 3E).

### 4. Co^2+^ Labeling in Retinal Culture with the Knockdown of ADAR2

**Figure 4:**
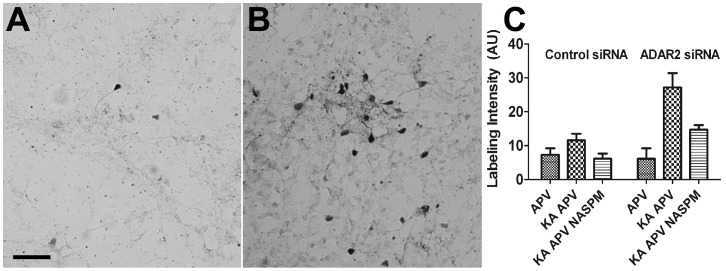
Co^2+^ labeling in retinal culture with the knockdown of ADAR2. Co^2+^ labeling was significantly increased when treated with KA and APV in cultures with ADAR2 siRNA, compared with scrambled siRNA (n = 7, *p*<0.005). The increase was reversed by NASPM, indicating Co^2+^ entered through CP-AMPARs (n = 7, *p*<0.05).

One functional means to test for an increase in the expression of CP-AMPAR is through the measurement of Co^2+^ permeability in cells of interest [23], [24]. While Co^2+^ cannot permeate through edited GluA2 containing receptors it can pass through CP-AMPARs. Retinal cultures were bathed in 5 mM CoCl_2_ with 20 µM kainate acid (KA), and 50 µM APV (20 min). KA activates AMPA receptors without causing desensitization and APV blocks NMDA receptors, preventing entry of Co^2+^ through this channel. Some preparations included NASPM (1-naphthyl acetyl spermine), a specific blocker of CP-AMPARs, to ensure any detected Co^2+^ entry was through this population of receptors. The cells were washed in an EDTA containing solution and the Co^2+^ was precipitated with (NH_4_)S followed by fixation, silver enhancement, and mounting. We found that Co^2+^ labeling was significantly increased when treated with KA and APV in cultures with ADAR2 siRNA, compared with scrambled siRNA. The increase of Co^2+^ precipitate was reversed by NASPM, indicating Co^2+^ entered through CP-AMPARs (Fig. 4).

### 5. Knockdown of ADAR2 Decreases the Rectification Index of AMPAR Currents

**Figure 5:**
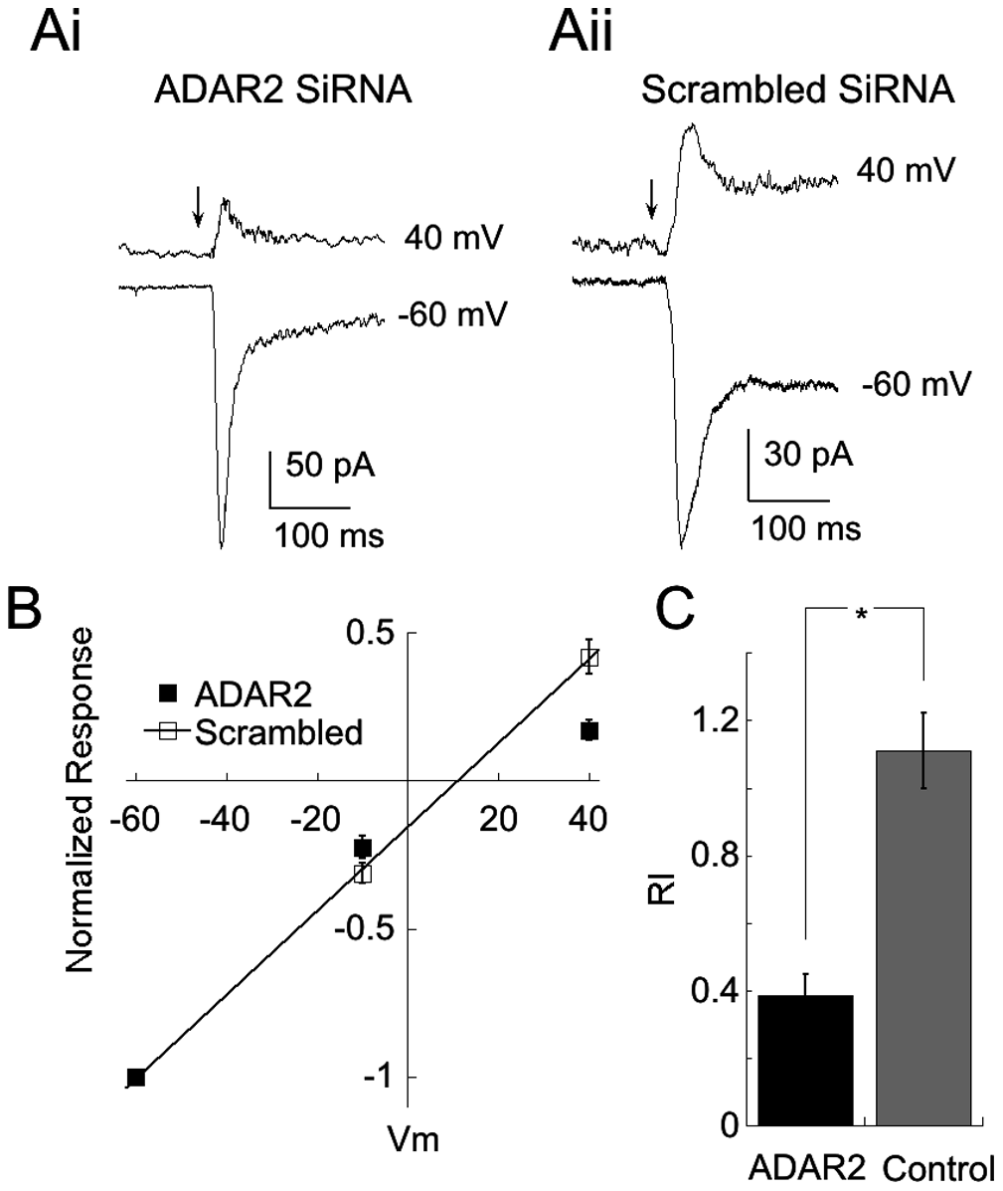
Knockdown of ADAR2 increases rectification of AMPAR currents. (A) Representative traces from an RGC transfected with ADAR2 SiRNA (Ai) and another transfected with scrambled SiRNA (Aii). Both cells were recorded from with a pipet solution containing 300 µM spermine, which blocks CP-AMPAR currents at positive, but not negative voltages. Responses to 50 ms puffs of AMPA (arrow) at −60 mV have been adjusted to approximately equal size so that the larger response to AMPA at 40 mV in the RGC transfected with scrambled SiRNA is apparent. (B) Summary I–V relation for ADAR2 (filled symbols, n = 14 cells) and scrambled SiRNA (open symbols, n = 12 cells). Responses in both groups have been normalized to the responses at −60 mV for each cell. Continuous line is fitted to the mean amplitude of the AMPA current at −60 and −10 mV for cells transfected with scrambled SiRNA. (C) Data from (C) replotted as the rectification index (RI), calculated as described in the Methods. The smaller number indicates a greater degree of rectification for RGCs transfected with ADAR2 SiRNA as compared to RGCs transfected with control, scrambled SiRNA (*indicates p<0.01). The data are consistent with the idea that knockdown of ADAR2 with SiRNA increases expression of CP-AMPARs in RGCs.

At positive voltages, CP-AMPA receptors are blocked by intracellular polyamines such as spermine, resulting in a strong inward rectification of AMPAR current [25]. CI-AMPARs are not effected by polyamines, thus providing an additional tool to distinguish between the two AMPAR populations. Accordingly we recorded from presumed RGCs, identified using previously established criteria [26], and applied AMPA (200 µM) through a second pipet using brief pulses (50–100 ms duration) of positive pressure. When spermine (300–500 µM) was included in the pipet solution, RGCs that had been transfected with ADAR siRNA 4–6 days earlier displayed significant inward rectification, as the response to AMPA at 40 mV was considerably smaller than predicted based on a linear I–V relation for AMPAR current (Fig. 5Ai, B). Conversely, RGCs transfected with scrambled siRNA displayed larger AMPAR currents at 40 mV, and a linear I–V relation (Fig. 5Aii, B). To quantify the difference in rectification between the two groups, we calculated the rectification index (see methods). The difference was highly significant (Fig. 5, C; ADAR2 siRNA, RI = 0.38±0.06, n = 14; scrambled siRNA, RI = 1.11±0.11, n = 12; p<.01). We conclude that under these culture conditions, RGCs normally express predominantly CI-AMPARs, but when ADAR2 levels are reduced, expression is dominated by CP-AMPARs.

### 6. TUNEL Staining

**Figure 6:**
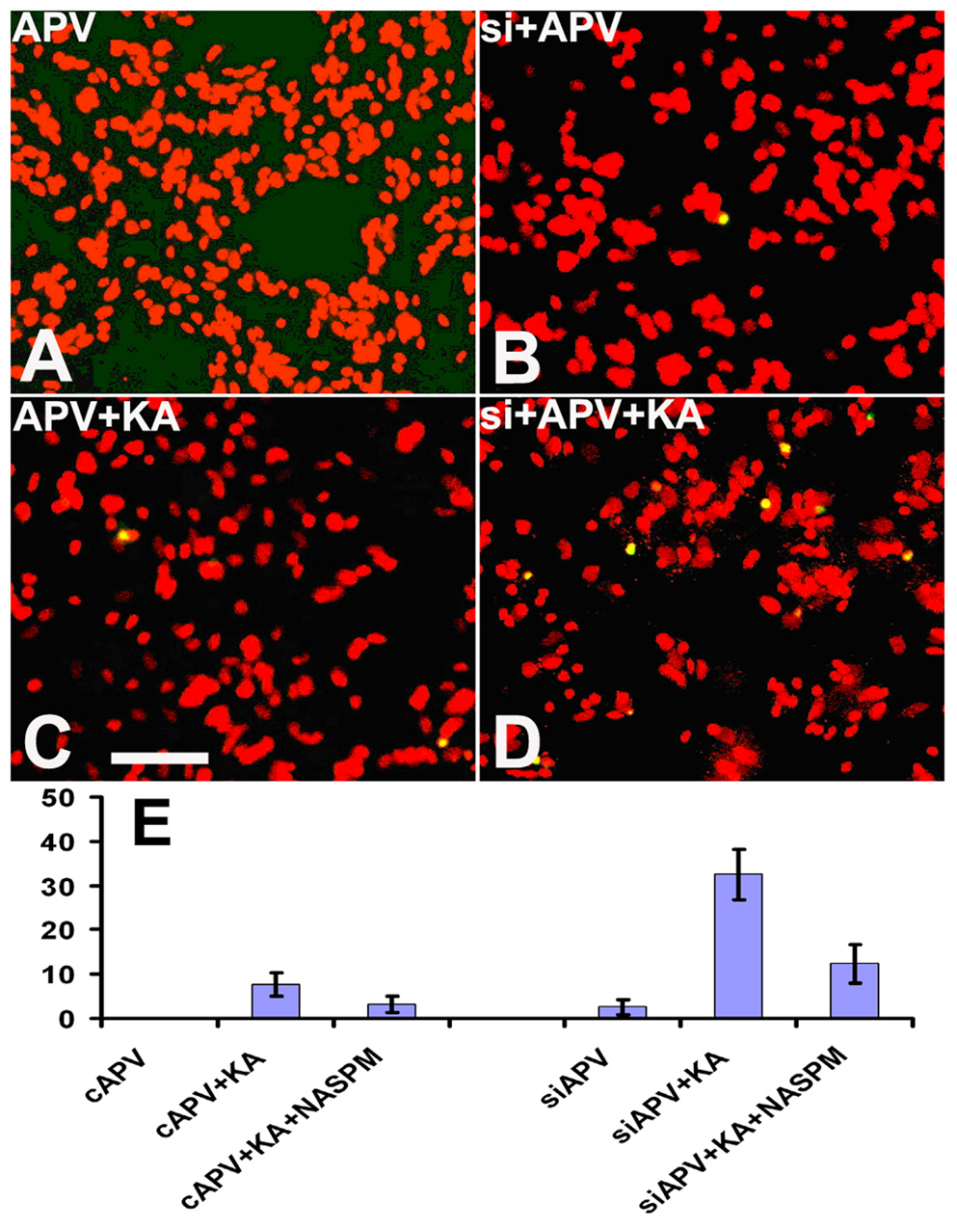
TUNEL staining. (A–D): TUNEL staining. Red: Propidium iodide (PI). Green: TUNEL positive cells. (A): APV control siRNA; (B): APV+ ADAR2 siRNA; (C): APV+KA control siRNA; (D): APV+KA ADAR2 siRNA. (E): Statistical analysis showed dramatically increased TUNEL positive cells in the condition treated with APV+KA in the knockdown group of ADAR2 siRNA (n = 3, p<0.001). KA: glutamate agonist. NASPM: 1-naphthyl acetyl spermine (a specific blocker of CP-AMPARs).

To evaluate if increased susceptibility of RGCs to excitotoxic cell death is associated with reductions in ADAR2, apoptosis was detected with TUNEL staining under the following conditions: scrambled siRNA+APV, scrambled siRNA+APV+KA, siADAR2+APV, siADAR2+APV+KA. The proportion of TUNEL positive cells was calculated in nine randomly selected images per condition. Abundant TUNEL^+^ cells were detected in the condition of siADAR2+APV+KA (Fig. 6D), while only a small number of apoptotic cells were detected in other conditions (Fig. 6A, B, C and D). Statistical analysis showed dramatically increased TUNEL positive cells in the condition treated with APV+KA in the knockdown group of ADAR2 siRNA compared with control siRNA (Fig. 6E; p<0.001).

## Discussion

Our study demonstrates for the first time that ADAR2, the enzyme responsible for GluA2 mRNA Q/R site editing, decreases in a chronically elevated IOP mouse model. We also find that RGCs in primary culture exhibit increased Ca^2+^ influx and cell death following knock down of ADAR2 using siRNA. Furthermore, cell death is reversed by NASPM, a specific blocker for CP-AMPARs. Together, our data suggest a sequence of events in which elevated IOP result in a loss of ADAR2 levels, a decrease in GluA2 Q/R site editing, an elevation in the proportion of CP-AMPARs, increased Ca2+ influx, and finally RGC cell death. Thus far we have demonstrated two of these steps, namely an increase in both CP-AMPAR expression and Ca^2+^ influx, in primary culture. These findings will need to be extended to the elevated IOP mouse model.

### Might CP-AMPAR be a Better Candidate than NMDAR for Contributing to RGC Death in Glaucoma?

A misconception has been that, for excitotoxicity to occur, elevated glutamate levels must be detected in the retina/ocular fluids of experimental animals or humans with glaucoma [27], [28]. Excess glutamate release is a hallmark of acute brain injuries with fast and severe neural tissue damage [29], but this is not necessarily characteristic of a slow, progressing neurodegenerative disease such as glaucoma. In retinal diseases with a clear ischemic component, such as retinal detachment, there is a detectable glutamate elevation in the vitreous and the subretinal space [30], [31]. However, the chronic, gradual nature of glaucoma defies any drastic elevation or accumulation of glutamate throughout the course of the disease. Another misconception is that the traditional excitotoxicity model for glaucoma focuses almost exclusively on the NMDAR. Antagonists of NMDAR have been intensely investigated as agents that may confer neuroprotection, but these compounds have consistently failed to show neuroprotection in human clinical trials [18], [32].

Accumulating evidence supports that the AMPA-type channels, are key contributors to neuronal injury [20]. NMDAR and AMPAR are two main kinds of receptors activated by glutamate. These two receptors-each with distinct physiological properties-often co-exist at the same synapse. Weak electrical stimulation of the presynaptic neuron causes the release of glutamate from the pre-synaptic terminal. Although both receptors are permeable to Na^+^ and K^+^ ions, weak stimulation normally activates only the AMPARs, resulting in a slight depolarization of the post-synaptic neuron. When glutamate binds to the NMDAR at slightly depolarized or resting membrane voltages, very few ions flow through the channel. This low conductance occurs because the pore of the channel is blocked by Mg^2+^ ions, which prevents other ions from passing freely through the channel [33]. Under such conditions, the EPSP will be mediated entirely by the AMPARs. Therefore, the NMDA ion channel only opens when the following two conditions are met simultaneously: glutamate is bound to the receptor, and the postsynaptic cell is depolarized (which removes the Mg^2+^ blocking the channel). From channel properties, at the same glutamate levels, AMPAR is more easily to be activated than NMDAR.

AMPAR channels are tetrameric complexes composed of various combinations of four subunits (GluA1–A4) [34], each encoded by a different gene. The AMPARs permeability to Ca^2+^ is governed by the GluA2 subunit. If an AMPAR lacks a GluA2 subunit, then it will be permeable to Ca^2+^. The presence of a GluA2 subunit will almost always render the channel impermeable to Ca^2+^ [8]. This is determined by post- transcriptional modification-RNA editing of the Q/R editing site of the GluA2 mRNA. Here, editing alters the uncharged amino acid glutamine (Q), to the positively-charged arginine (R) in the receptor’s ion channel. The positively-charged amino acid at the critical point makes it energetically unfavorable for Ca^2+^ to enter the cell through the pore. Therefore, the AMPAR’s permeability to Ca^2+^ is governed by the edited GluA2 subunit. If an AMPAR lacks an edited GluA2 subunit, then it will be permeable to Ca^2+^ (CP-AMPAR). The presence of an edited GluA2 subunit will almost always render the channel impermeable to Ca^2+^ (CI-AMPAR). Ca^2+^ influx through CP-AMPARs is crucial in several forms of synaptic plasticity and has also been linked to cell death associated with neurological diseases [21].

Here, we present evidence that Ca^2+^ entry in RGCs through CP-AMPAR is sufficient to activate pro-apoptotic signaling cascades in RGCs. and that a role for NMDARs is not required. The Ca^2+^ permeability of this receptor is much lower than the NMDA receptor. However, over time, the influx of Ca^2+^ through even a mildly Ca^2+^-conducting channel would be expected to overwhelm the buffering capacity of an RGC. In fact, the slow time course of excitotoxicity that is predicted by the CP-AMPAR model, compared with the NMDA receptor model, is more consistent with the observed time course in glaucoma patients. This scenario may lead to CP-AMPAR channel opening and excitotoxic damage even at physiological levels of glutamate.

### Down-regulation of ADAR2, an Editing Enzyme for GluA2, Might be a Common Factor for Glaucoma and other Neurological Diseases

As mentioned above, AMPARs that contain the GluA2 subunit are impermeable to Ca^2+^. However, this requires that the GluA2 transcript is edited at the Q/R site, resulting in substitution of an arginine residue for glutamine [26]. The enzymes responsible for RNA editing are termed “adenosine deaminases acting on RNA” (ADARs), and three structurally related ADARs (ADAR1 to ADAR3) have been identified in mammals [35]. ADAR1 and ADAR2 are widely detected in various tissues, with strong expression in the brain. ADAR2 predominantly catalyzes RNA editing at the Q/R sites of GluA2 both *in* *vitro* and *in* *vivo*, whereas both ADAR1 and ADAR2 catalyze the Q/R sites of GluR5 and GluR6 subunits of kainite receptors. ADAR3 is detected only in the brain, but its deaminating activity has not been demonstrated. Thus, injury or diseases that result in decreased expression of the ADAR family could be expected to result in increased Ca^2+^ influx into neurons through AMPARs that are normally impermeable to Ca^2+^. ADARs are essential in mammals and are particularly important in the nervous system. Altered levels of ADAR2 editing are observed in several diseases.

In motor neurons of sporadic amyotrophic lateral sclerosis (ALS) patients expression levels of ADAR2 is reduced and neuronal death has been attributed to increased Ca^2+^ permeability through the unedited GluA2-containing AMPA receptors [36]. ADAR2 editing efficiency, among the motor neurons of each individual with sporadic ALS, was not complete in 44 of them (56%), whereas it remained 100% in normal controls. Because ADAR2 specifically catalyzes GluA2 Q/R editing, it is likely that ADAR2 activity is not sufficient to edit this site completely in motor neurons of patients with sporadic ALS. Because these molecular abnormalities occur in disease- and motor neuron-specific fashion and induce fatal epilepsy in mice, GluA2 Q/R under-editing due to ADAR2 under-activity has been proposed to be a cause of neuronal death in sporadic ALS. The restoration of this enzyme activity in ALS motor neurons may open the novel strategy for specific ALS therapy.

A molecular explanation for why some neurons are more vulnerable than others to ischemic injury has long remained elusive. Recent studies showed forebrain ischemia in adult rats selectively reduces expression of ADAR2 enzyme and disrupts RNA Q/R site editing of GluA2 subunit in vulnerable neurons [37]. Recovery of GluA2 Q/R site editing by expression of exogenous ADAR2b gene or a constitutively active CREB, VP16-CREB, which induces expression of endogenous ADAR2, protects vulnerable neurons in the rat hippocampus from forebrain ischemic insult. Generation of a stable ADAR2 gene silencing by delivering siRNA inhibits GluA2 Q/R site editing, leading to degeneration of ischemia-insensitive neurons. Direct introduction of the Q/R site edited GluA2 gene, rescues ADAR2 degeneration. Thus, down-regulation of ADAR2, resulting in defective Q/R editing of GluA2 and increased availability of Ca^2+^-permeable death-promoting AMPA receptors, underlies the vulnerability of some neuronal populations to ischemia.

Schizophrenia and bipolar disorder (BPD) are common neurodevelopmental disorders, characterized by various life-crippling symptoms and high suicide rates. Altered GluA2 RNA editing and ADAR2 splice variants have been proposed as a molecular mechanism underlying the disorder [38]. Thiamine deficiency leads to mild impairment of oxidative metabolism and region defective neuronal loss in the central nervous system. Deficiency of thiamine has been shown to cause alterations in editing of GluA2 pre-mRNA. Deficiency also inhibits editing of the Q/R site of GluA2 thereby increasing the level of unedited GluA2 resulting in increased Ca^2+^ permeability of GluA2 containing channels [39].

In summary, these data suggest that altered ADAR2 activity may be a common mechanism in several neuronal diseases. Ca^2+^-permeable AMPARs and their RNA processing machinery ADARs may be promising therapeutic targets for intervention in these neurological diseases. Furthermore, our data provide evidence that ADAR2 and CP-AMPARs may contribute to RGCs dysfunction due to elevated Ca^2+^ influx in glaucoma. Restoring of ADAR2 may be a novel target for the therapeutic intervention in glaucoma.
